# Struggling With Business Corporate Cynical Impression? Powerful Methods of CSR to Enhance Corporate Image and Consumer Purchase Intention

**DOI:** 10.3389/fpubh.2021.726727

**Published:** 2021-09-03

**Authors:** Andrianarivo Andriandafiarisoa Ralison Ny Avotra, Ye Chengang, Xu Wei, Jiang Ming, Tsimisaraka Raymondo Sandra Marcelline

**Affiliations:** ^1^Business School, Zhejiang Wanli University, Ningbo, China; ^2^Business School, University of International Business and Economics, Business School, Beijing, China

**Keywords:** economies CSR, business population, corporate cycle, population health, corporate capital

## Abstract

This study focuses on the perception of Chinese students about the image of the company and their purchase intention if the organization has a business cynical impression in the minds of its targeted customers. The study proposed three different types of corporate social responsibility (CSR) to cope up with the organizational cynical impression. These types are Economic and Legal CSR, Philanthropic CSR, and Ethical CSR. The main objective of this study is to determine which CSR type is better to reduce the corporate cynical impression on corporate image and the purchase intentions of consumers. In the study design, the bootstrap approach and AMOS 24 were employed to deal with mediation. The researchers recruited 500 individuals from different educational institutions in China using a simple random selection process. The outcomes of this study indicated that all three types of CSR are successful in mitigating the detrimental effects of corporate cynicism on the image of a firm and the purchase intentions of consumers. A more effective technique of boosting the corporate image of a company and purchase intention of a consumer is *via* charitable CSR, which may help restore the image of a company and the purchase intention of a consumer that has been affected by corporate cynicism among its target customers.

## Introduction

Environment competition in the current business has exploded in every aspect of life. This cut-throat competition at workplaces gives rise to cynicism factors such as jealousy and other negative attitudes toward the organization (1). Organizational cynicism is a pessimistic approach and originates when employees perceive that the company lacks integrity, which is usually the consequence when the employees are not meeting their basic expectations such as ethics, honesty, and justice. Organization can reduce their cynicism characteristics in employees by improving their corporate image through the utilization of proper corporate social responsibility (CSR) activities (2).

Corporate social responsibility in the current decades is becoming more important and growing interest has attracted the attention of stakeholders and scholars. It is a management approach that entails the organizations to incorporate social and environmental concerns in their business activities and interactions with the stakeholders. This motivated the companies to conduct diverse CSR activities as they realized both intangible and tangible aspects are compulsory for conducting business. Furthermore, socially responsible activities are becoming increasingly important to all stakeholders. Thus, corporations can enhance their relationships with stakeholders by adopting different CSR strategies (3). In achieving this, corporations should communicate their CSR activities efficiently with their stakeholders by developing management relationships to meet their expectations and accomplish the desired goals of CSR projects (4).

With rising globalization and strong rivalry in the business, CSR is now one of the most important components of an organization to create long-term sustainability and competitive advantage. A properly implemented CSR framework adds a lot of competitive advantages to an organization, such as enhanced corporate image, better risk management, improved quality and productivity, increased sales, and profit maximization. Furthermore, the framework helps in decision-making processes, enhances loyalty and the purchase intentions of consumers (5). CSR has been studied from different perspectives while widely recommended CSR components include legal, economic, philanthropic, and ethical CSR. Many previous studies have investigated the effect of CSR on different organizations (6–8).

Overall global companies are attempting to respond their all stakeholders, i.e., suppliers, consumers, government, and communities, through adopting CSR activities. Consumers are one of the most important stakeholders of an organization and becoming more aware of incorporating CSR activities into their purchasing decision. Empirical evidence concluded that the attitudes and intentions of consumers are influenced by CSR activities only if consumers have awareness related to such activities (9). Furthermore, organizations can enhance their corporate image by utilizing CSR activities efficiently which in turn has a significant impact on the purchase intention of their consumer. Related works of literature also revealed that CSR activities can improve the attitudes and purchase intentions of the consumers, as well as the corporate image (10–12).

Existing studies have demonstrated the impact of organizational cynicism in other different contexts. To the best of our knowledge, organizational cynicism has not been studied with CSR initiatives in such a way that how the organizations can improve their image and consumer purchase intentions through mediating the role of CSR (economic, legal, ethical, and philanthropic) initiatives (13–15).

The remaining structure of the study is as follows: the second section provides related works of literature to review prior studies on considered constructs. The third section discusses the methodology of the study which was employed to test the hypothesis. The fourth section is related to the interpretation of our empirical study. Lastly, the fifth section concludes the study by offering implications and future recommendations.

## Literature Review

### Organization Cynicism

Organizational cynicism has been defined in works of literature from different perspectives but researchers have identified certain fundamental challenges in conceptualizing organizational cynicism. According to the study conducted by Crisostomo, (16) organizational cynicism has been defined as the negative attitude and judgment of an employee toward the processes and structure of their organization. On the other hand, organizational cynicism is termed as the negative emotions of a person toward their employment and the beliefs that the organization lacks in integrity and honesty. Organizational cynicism involves negative emotional reactions and sharp criticism toward the workplace. Previous studies concluded various factors which highlight cynicism in organizational stakeholders, such as administrative competence, organizational support, working environment, and trust of administration (17). Based on the prior discussion, we can propose our hypothesis as follows.

H^1^: Corporate cynical impression has a negative impact on the corporate image.
H^2^: Corporate cynical impression has a negative impact on the purchase intention of the consumer.

### Corporate Social Responsibility

Many authors argued that CSR does not have a universally accepted definition and has been defined from different perspectives. Most definitions of CSR consist of components such as environmental, social, and all stakeholders of the company (18–20). CSR comprises ethics, business relationships, governance, community involvement, transparency, and many other employment practices. It has been found that CSR and its components significantly increase economic benefits which motivate the organizations to adopt those CSR activities to help them in developing strategies. Furthermore, CSR is a voluntary commitment of corporations to transcend implicit and explicit obligations which are imposed on corporations by the expectations of society of traditional corporate behavior (21–24). CSR has main four dimensions: economic, legal, ethical, and philanthropic activities. Studies suggested that economic and legal CSR activities are socially required, ethical CSR initiatives are socially expected, and philanthropic CSR activities are socially desired (25–27).

### Economic and Legal CSR

Economic CSR is primarily concerned with the economic development and progress of society and addresses the economic responsibilities of an organization to its stakeholders. The economic CSR comprises investment return for stakeholders and owners, creation of employment, remuneration for the employees, and discovery of new resources. Whereas legal CSR is concerned with rules and regulations which organizations must follow (28). It includes expectations, specific rules, and regulations as well as the role with respect to playing regulations (29, 30). Legislations, control, and regulations are essential drivers toward compliances and compulsory for the existing CSR standards. Due to this, societies expect businesses to consider the social-legal system while carrying out their economic purpose (31–34). Based on the above discussion, we can propose our hypothesis as follows.

H^3^: Economic and Legal CSR has a positive impact on the corporate image.
H^4^: Economic and Legal CSR has a positive impact on the purchase intention of the consumer.

### Philanthropic CSR

Philanthropic CSR activities are referred to as the involvement of a corporation in promoting human welfare and goodwill have a broad range for assessing and selecting decision-making situations for specific activities and humanitarian collaboration to benefit society (35). The origin of philanthropic CSR is that business and society are interwoven through inherent and fundamental approaches. Philanthropic activities are identified as an optional add-on, as a charitable obligation and voluntary action, these are the actions that create social and economic benefits and corporate wealth is being utilized for welfare and development purposes (36). Based on the prior discussion, we can propose our hypothesis as follows.

H^5^: Philanthropic CSR has a positive impact on the corporate image.
H^6^: Philanthropic CSR has a positive impact on the purchase intention of the consumer.

### Ethical CSR

The modern business environment nowadays is seeing an increase in demand for ethical CSRs (37, 38). Ethical CSR is related to those activities which are not specified by the law but are expected to be done by corporations for the respect toward individuals, prevent the loss, and minimizing societal damage (39, 40). These responsibilities have rooted in human rights. In simple words, ethical responsibilities are concerned with the obligation of the company to make fair decisions and successfully combine the interest of society. Many studies claim that companies are realizing that generating funds requires an ethical foundation (41, 42). Hence, ethical CSR is a powerful tool of differentiation and offers various benefits to companies, such as advocacy, purchase intentions, consumer loyalty, and building a positive corporate image (43–45). Based on the above discussion, we can propose our hypothesis as follows.

H^7^: Ethical CSR has a positive impact on the corporate image.
H^8^: Ethical CSR has a positive impact on the purchase intention of the consumer.

### Corporate Image

Corporate image has been demonstrated to be an important precursor to service and corporate evaluation. This is because the corporate image is a public perception of beliefs, information, experiences, emotions, and impressions of a company (46, 47). On the other hand, corporate image is defined as a non-physical component of a company and its image in the mind of stakeholders. These stakeholders which are both internal and external are essential elements of a company. Corporate image is linked to the characteristics of a company which are subdivided into CSR initiatives and corporate capability (48, 49). All positive characteristics of a company lead to a good corporate image which in turn increases the loyalty and purchase intentions of the consumer to buy the products of a company (50).

Corporate image is a valuable asset and provides a strategic advantage to an organization. The companies can attain a competitive advantage in the competitive market through a positive corporate image. Increasingly, organizations are emphasizing their CSR efforts which improve their corporate image. Previous studies addressed that corporations can shape their positive corporate image by public awareness of CSR activities (51–53).

### Consumers Purchase Intentions

Corporate social responsibilities have a significant impact on the willingness to pay and purchase intentions of a consumer (54). The purchase intentions of a consumer are influenced by local community contribution and corporate social contribution. The local community members establish their association with the organization and this bonding influences their purchasing intentions. Furthermore, many authors argued that consumers exhibited a higher purchase intention for socially responsible companies than the companies who are socially irresponsible (55, 56). Companies can enhance the purchase intentions of their consumers by taking CSR initiatives in organizations. Previous studies concluded that CSR activities have a positive impact on the purchase intentions of consumers. Therefore, companies should focus on CSR activities because these help organizations to enhance their corporate image, which will ultimately influence the purchase intentions of consumers (22, 25, 35, 36, 57).

### Organizational Cynicism and Corporate Image

Organizational cynicism is a pessimistic approach to the attitude of an individual at its workplace, it is also a belief that an organization is lacking its honesty, has a bad effect on its workplace, and tendency to disapprove significant activities against the firm that are reliable with these attitudes and effects (4, 11, 16, 58). Organizations can improve their positive image and can attract their consumers through CSR activities. The perception of consumers on the products and services of a company plays a significant role in developing a positive corporate image in the mind of an individual. Again, corporate image is defined as the perception of the public about beliefs, information, experiences, emotions, and impressions of a company. It has also been termed as a non-physical component of a company and its image in the mind of stakeholders. Companies can attain a competitive advantage in the competitive market through a positive corporate image (16, 34, 58–60). All positive characteristics of a company lead to a good corporate image which in turn increases the loyalty and purchase intentions of consumers to buy the products from a company (61). Based on the prior discussion, we can propose our hypothesis as follows.

H^9^: The negative impact of corporate cynical impression could be reduced upon corporate image when we introduce Economic and Legal CSR as mediators in this relationship.
H^10^: The negative impact of corporate cynical impression could be reduced upon corporate image when we introduce Philanthropic CSR as a mediator in this relationship.
H^11^: The negative impact of corporate cynical impression could be reduced upon corporate image when we introduce Ethical CSR as a mediator in this relationship.

### Organizational Cynicism and Consumers Purchase Intentions

Organizational cynicism is one of the factors which have a negative effect on the purchase intentions of consumers. Organizational image influences the purchase intentions of a consumer either directly or indirectly. Organizational negative characteristics badly influence the trust and the purchase intentions of consumers. On the other hand, organizational positive characteristics enhance corporate image and build a strong relationship with the consumers to purchase its products (24, 29, 48). Based on the above literature, we can propose our hypothesis as follows:

H^12^: The negative impact of corporate cynical impression could be reduced upon consumer purchase intention when we introduce Economic and Legal CSR as mediators in this relationship.
H^13^: The negative impact of corporate cynical impression could be reduced upon consumer purchase intention when we introduce Philanthropic CSR as a mediator in this relationship.
H^14^: The negative impact of corporate cynical impression could be reduced upon consumer purchase intention when we introduce Ethical CSR as a mediator in this relationship.

### CSR Dimensions and Organizational Cynicism

Organizational cynicism is termed as the negative emotions of a person toward their employment and their belief that the organization lacks in integrity and honesty. Organizational cynicism involves negative emotional reactions and sharp criticism toward the workplace. Organizations can improve their corporate image and can reduce the influence of negative factors through CSR activities (23, 33). CSR is considered an important factor for the success of an organization. It can be termed as a strategic approach that helps companies to reduce the negative impact of organizational cynicism on their image. CSR activities among organizations and their stakeholders often contribute to the macroeconomic progress of a developing nation by providing long-term benefits to all (1, 37, 46). Previous studies have investigated the impact of CSR initiatives on businesses and suggested that implementing socially responsible initiatives in organizations can result in a variety of positive eventual results (8, 47, 62). CSR is regarded as one of the best practices for avoiding the negative image of a company and gaining legitimacy. All these CSR initiatives including economic, legal, ethical, and philanthropic create a positive image of the corporation and reduce organizational cynicism (8, 15, 33, 62).

### CSR Dimensions and Corporate Image

Companies can improve their corporate image by engaging in CSR activities because a positive corporate image works as a competitive advantage for companies. More companies can attract the purchase intentions of their consumers through CSR activities and a positive corporate image (23, 27, 39), in addition it will provide enough light to CSR initiatives (economic, legal, ethical, and philanthropic) and their impact influencing corporate image. Economic CSR is the responsibility of a company to generate funds through economic activities, and it describes the maintenance of competitive advantage, maximizing of earning per share, and encountering continual profits which are the indicator of a successful business (49, 50, 53, 63). Legal CSR is concerned with doing business fairly and achieving or surpassing all relevant legal responsibilities at local and federal levels. The previous studies revealed that CSR activities have a significant positive impact on corporate image. Ethical responsibility is identified by minimization of risk and internal control system that can lead to the offering of products that are beneficial for consumers. Related works of literature have asserted the impact of unethical CSR and ethical CSR on the corporate image and found that ethical CSR enhances corporate image significantly (29, 43, 48). Philanthropic CSR corresponds to improving the quality of life for society as a whole by donating the resources of a company, facilities, and time to the community of an employee for free. The prior studies have documented that philanthropic CSR activities have an influential impact on brand attitude which leads to enhance a positive corporate image (30).

### CSR Dimensions and Consumer Purchase Intentions

**Figure 1:**
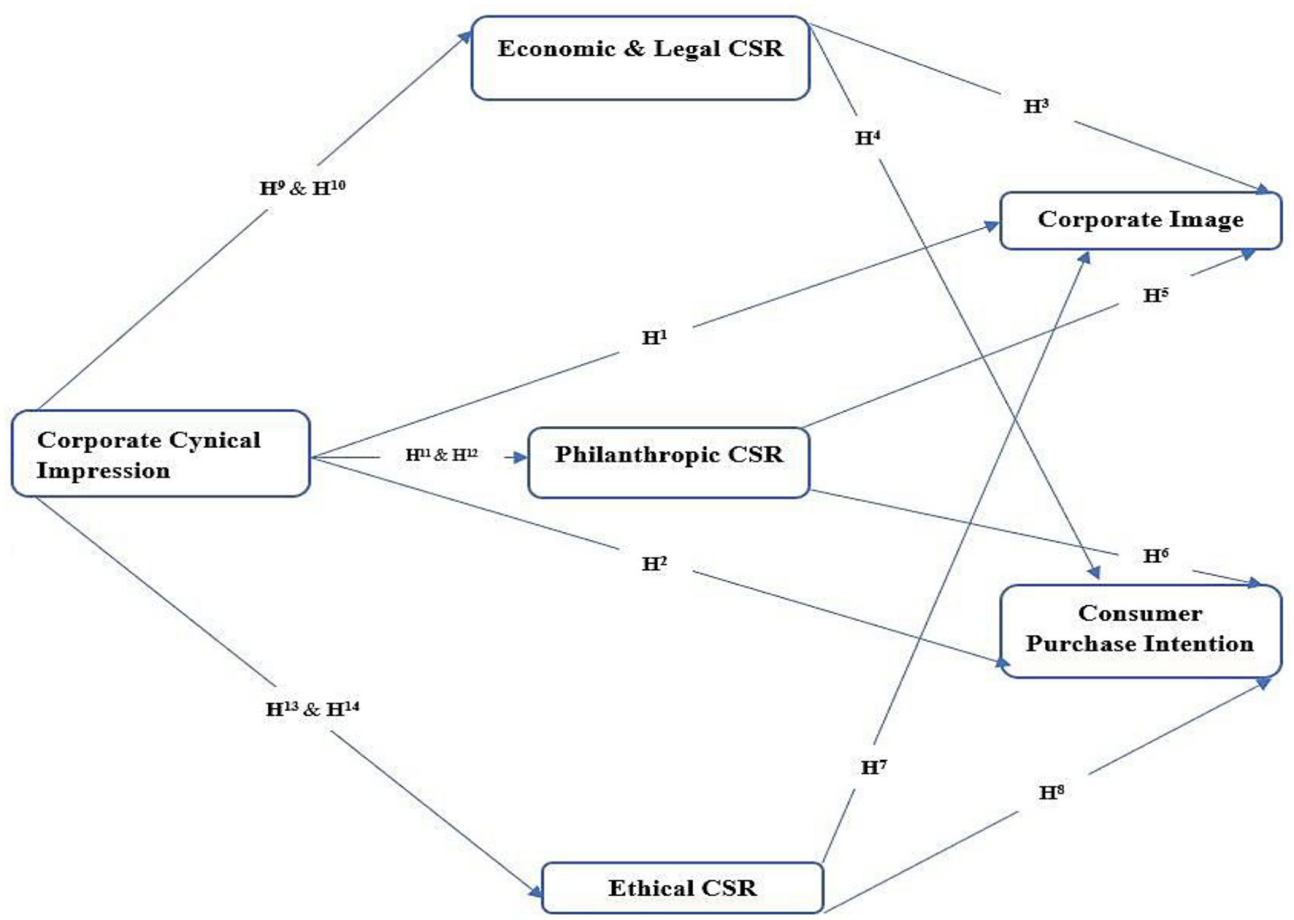
Theoretical framework.

In general, CSR initiatives significantly affect the behavior and intention of a consumer to purchase the products of a company (59). Specifically, CSR information influences the attitude and the purchase intentions of a consumer toward the products of a company (62). It has been argued that companies can attain a competitive advantage on the purchase intention of consumers through CSR activities (17, 19, 41). This study proposed how CSR dimensions, which are economic, legal, ethical, and philanthropic, affect the purchase intentions of consumers. Economic CSR is primarily concerned with the economic development and progress of society (3, 29, 43, 48). According to economic responsibility is a source of long-term competitive advantage while its strategic perspective is to focus on wealth generation and profit maximization. It has been found that consumers who believe that the corporation is socially responsible are more likely to purchase its products (23, 33, 39, 47). Economic and legal CSR has a positive impact on consumer buying behavior and their intentions to buy products of socially responsible firms. Philanthropic CSR has a positive impact on consumer buying behavior and their intentions to buy products of socially responsible firms. Ethical CSR is the obligation of businesses to execute best practices demanded by society members, without relying on legal laws. These practices are the indicator of the healthy social traditions, ethical compliance, and excellent corporate citizenship of a company. The ethical CSR of a company leads to a positive impact on the attitude and purchase intention of a consumer (16, 25, 34, 36). The theoretical framework of this study shows the workflow of the study with hypothesis development (see Figure 1).

## Research Methodology

The methodology used in a study is a critical and fundamental aspect of the whole process since it illustrates how science helps to the achievement of objectives and goals. The analysis parameter can be summarized by expectations, evidence, observations, knowledge of reality, views, and studies of the behavior of respondents in general. It is also needed to ensure objectivity in science (55, 64). Furthermore, it is vital to provide a perspective that emphasizes social variables and the need to consider society (65). Two main activities are treated under the label of research, which is discovery and interpretation.

### Research Design

Related works of literature describe behavioral science overview (5, 34, 35). In this process, mediation and description work together to solve the problem. Studies may concentrate on states of mind, mood, feelings, or behaviors (16). It developed a cooperative feedback process with its peers and gathered knowledge about itself. An interesting analysis is when applied to two or more distinct variables such as creative methods (66, 67). It can answer all questions because of its ability to be applied to any variable or how an organization performs in the market and impacts consumer purchase intention (58). Self-administered questionnaires are used in quantitative analysis for data collection.

### Study Population

The present research focuses on students from several Chinese institutions. The primary rationale for picking university students as a group is that a recent study has focused on business cynicism concerning the purchase intention of a consumer and business image. The setting of this study also uses the said population due to their interest and importance while considering the objectives of this study. The data is collected through various online and offline resources. The questionnaire is sent to 500 students; among them 357 responded, making the response rate of 73%. Among these 357 questionnaires, almost seven questionnaires are rejected based on incomplete information, making the final valid count 350 and the response rate 70%. The study uses convenience sampling for data collection. The objective of this study is to determine the effect of corporate cynicism on consumer purchase intention and business image while accounting for all three categories of CSR as a mediating variable. Few authors advised that a questionnaire be used during field examinations (64, 65). The researcher collected data in this study *via* the use of a questionnaire. The quality, validity, and scale reliability of the instrument were evaluated using the statistical tool IBM-SPSS vs 25.

## Data Analysis

Data analysis is performed using the Statistical Package for the Social Sciences (IMB-SPSS 25) and IBM-SPSS-AMOS software packages. The reliability analysis is shown in Table 1. In this research, corporate cynicism is an independent variable, while corporate image and consumer purchase intention are dependent variables. All three categories of CSR were shown to be mediating in this research. Each variable has a satisfactory reliability alpha value.

**Table 1:** Reliability analysis.

**Variables**	**Items**	**Cronbach's alpha value**
Corporate cynical Impression	3	0.747
Economic & Legal CSR	4	0.713
Philanthropic CSR	3	0.814
Ethical CSR	3	0.768
Corporate Image	4	0.742
Consumer Purchase Intention	4	0.767

The values in Table 2 are shown below. Corporate cynicism and CSR measures have an *M* value of 3.4, whereas economic and legal CSR has a mean value of 3.9, Philanthropic CSR has an *M* value of 4, and Ethical CSR has an *M* value of 3.8.

**Table 2:** Descriptive statistics.

**Variables**	**Mean**	**Std. deviation**	***N***
Corporate cynical Impression	3.4	0.93455	350
Economic & Legal CSR	3.9	0.98643	350
Philanthropic CSR	4.0	0.65432	350
Ethical CSR	3.8	0.75389	350
Corporate Image	3.2	0.76539	350
Consumer Purchase Intention	3.7	0.87654	350

### Instrument

**Figure 2:**
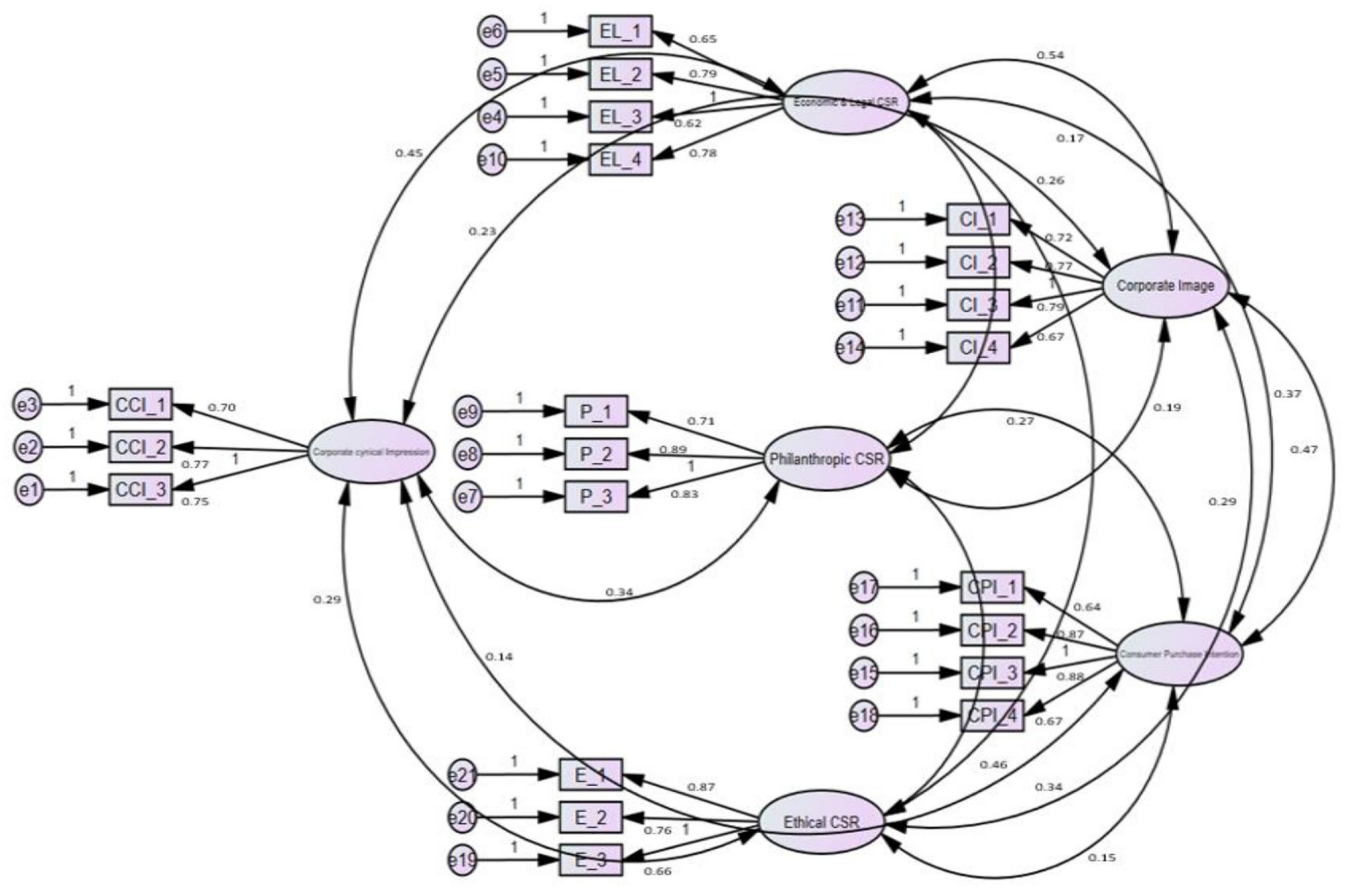
Pooled CFA.

For measuring corporate cynical impression, a five-point Likert scale ranging from strongly agree to strongly disagree has been adopted, which has been validated by confirmatory factor analysis (CFA) to meet the objectives of the study. To measure the concept of Economic and Legal CSR, Philanthropic CSR, Ethical CSR, and Corporate Image, we will use the scale developed by Wilcox and Iglesias studies (2, 25). While for measuring consumer purchase intention we will use a scale (5, 25, 34, 57), it was slightly modified by the authors to fit the scope of this study and then verified by the CFA methodology (see Figure 2).

### Confirmatory Factor Analysis

Pooled CFA is the latest and more reliable technique. In this methodology, the AMOS 24 runs all latent variables simultaneously (see Table 3).

**Table 3:** Pooled confirmatory factor analysis (CFA) model fitness tests.

**Name of Category**	**Name of index**	**Index full name**	**Value in analysis**	**Acceptable value**	**References**
Absolute Fit	RMSEA	Root Mean Square of Error Approximation	0.055	<0.80	(34)
Incremental Fit	CFI	Comparative fit index	0.931	>0.90	(58)
Parsimonious Fit	Chisq/df	Chi-Square / Degrees of freedom	1.432	<3	(32)

Pooled Table 4 shows the reliability value or factor loading of every item separately. It also shows the composite reliability of a complete scale of any variable. The reliability of the measurement scales was measured with composite reliability, which is preferred to report the reliability of a scale, a widely used indicator.

**Table 4:** Factor loading of items.

**Scale**	**Items**	**Factor loadings**	**Scale reliability**
Corporate cynical Impression	I do not like to do business with that organization which have negative impression.	0.707	0.747
	I do not like to do business with that organization which do not have good will and is not trustworthy.	0.777	
	I do not like to do business with that organization which did not have a better reputation than the other companies.	0.758	
Economic & Legal CSR	I would like to do business with that organization which can guarantee investor benefits.	0.654	0.713
	I would like to do business with that organization which can enhance corporate operational performance.	0.790	
	I would like to do business with that organization which can make public its information such as financial conditions and performance in good faith.	0.628	
	I would like to do business with that organization which can abide by the law and pay taxes honestly.	0.783	
Philanthropic CSR	I would like to do business with that organization which sponsor artistic and cultural events.	0.719	0.814
	I would like to do business with that organization which sponsor charitable groups.	0.890	
	I would like to do business with that organization which emphasize and accommodate charitable and public welfare events.	0.834	
Ethical CSR	I would like to do business with that organization which emphasize employee education and development.	0.871	0.768
	I would like to do business with that organization which save energy and reduce waste of resources.	0.766	
	I would like to do business with that organization which devote itself to protecting personal data of customers.	0.668	
Corporate Image	I would like to do business with that organization which have better impression.	0.729	0.742
	I would like to do business with that organization which has good will and is trustworthy.	0.773	
	I would like to do business with that organization which has a better reputation than the other companies.	0.798	
	I would like to do business with that organization which has a good overall image.	0.671	
Consumer Purchase Intention	I will purchase products of that organization which significantly perform CSR activities.	0.641	0.767
	I desire to buy products of that organization which significantly perform CSR activities.	0.873	
	I am likely to buy products of that organization which significantly perform CSR activities.	0.881	
	I plan to purchase products of that organization which significantly perform CSR activities.	0.674	

### Assessment of Discriminant Validity

Convergent validity is a subtype of construct validity that is defined as follows: the term “construct validity” refers to the fact that a test is meant to assess if a certain construct, e.g., IQ is really assessing that particular construct (68, 69). Convergent validity is the ability to demonstrate that two measurements that are meant to assess the same concept are really assessing the same phenomena (70–72). On the other hand, discriminant validity indicates that two measurements that are not meant to be associated are in fact not associated. Excellent construct validity requires the presence of both forms of validity (73). The heterotrait-monotrait ratio of correlations (HTMT) analysis was used to determine discriminant validity, with the cut-off threshold for severe discriminant validity being 0.850 and for liberal discriminant validity being 0.900. The values in Table 5 show that the items fulfill the criteria of the discriminant validity.

**Table 5:** Heterotrait-monotrait ratio of correlations (HTMT) analysis.

	**Corporate cynical Impression**	**Economic & legal CSR**	**Philanthropic CSR**	**Ethical CSR**	**Corporate image**	**Consumer purchase intention**
Corporate cynical Impression	X					
Economic & Legal CSR	0.485	x				
Philanthropic CSR	0.362	0.320	x			
Ethical CSR	0.307	0.285	0.281	x		
Corporate Image	0.285	0.197	0.167	0.134	X	
Consumer Purchase Intention	0.262	0.185	0.112	0.101	0.097	X

### Assessment of Discriminant Validity

The postulated relationships are investigated in this work using structural equation modeling (SEM). This analysis includes exogenous factors to facilitate the examination of endogenous variables through AMOS 24. In this study, independent and dependent variables can be seen as linearly related to one another. The basic design was constructed by using observed facts to build on (65), all observations were tabulated and linked to information on their mean values for analysis. Table 6 shows the model fit indices for the structural model, and it shows that they are meeting the acceptance criteria.

**Table 6:** Structural equation modeling (SEM), model fitness tests.

**Name of Category**	**Name of index**	**Index full name**	**Value in analysis**	**Acceptable value**	**References**
Absolute Fit	RMSEA	Root Mean Square of Error Approximation	0.056	<0.80	(26)
Incremental Fit	CFI	Comparative fit index	0.915	>0.90	(18)
Parsimonious Fit	Chisq/df	Chi Square/Degrees of freedom	2.919	<3	(39)

The following Table 7 illustrates the direct influence of the independent variable on the dependent variable.

**Table 7:** Results of direct effects.

**Hypothesis**	**Causal Path**	**Lower Bound**	**Upper Bound**	***P*-Value**	**Standardized Estimated**
***H**^**1**^*	Corporate cynical Impression → Corporate Image	−0.162	0.093	0.001	0.23
**H** ^**2**^	Corporate cynical Impression → Consumer Purchase Intention	−0.183	0.026	0.045	0.17
**H** ^**3**^	Economic & Legal CSR → Corporate Image	−0.162	0.093	0.034	0.12
**H** ^**4**^	Economic & Legal CSR → Consumer Purchase Intention	−0.183	0.026	0.030	0.20
**H** ^**5**^	Philanthropic CSR → Corporate Image	0.096	0.378	0.005	0.35
**H** ^**6**^	Philanthropic CSR → Consumer Purchase Intention	0.219	0.464	0.003	0.43
**H** ^**7**^	Ethical CSR → Corporate Image	0.167	0.345	0.023	0.19
**H** ^**8**^	Ethical CSR → Consumer Purchase Intention	0.176	0.256	0.042	0.25

**Figure 3:**
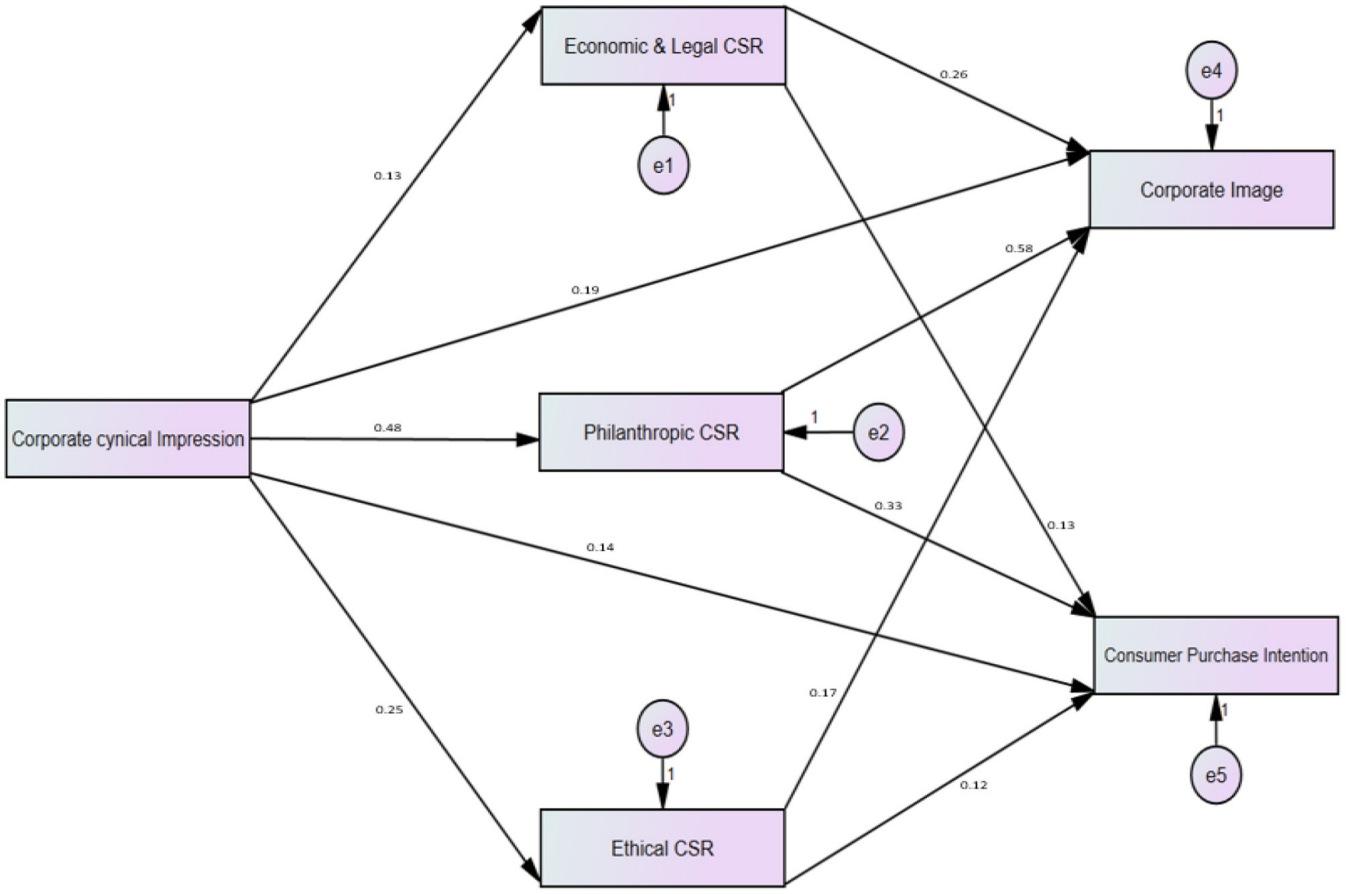
Indirect direct effects of path analysis.

Table 7 shows that all the hypotheses are statistically significant and their *p* <0.05 which shows the confidence interval of 95%. While Figure 3 shows the path analysis of structural equation modeling. In this path analysis, all six variables are present in which one variable is independent, three are mediators, and one dependent variable is present (see Figure 3).

According to Table 8, all six hypotheses are statistically significant, and the observed mediation for these hypotheses is classified as partial mediation. Full mediation implies that the mediating variable explains the whole link between the independent and dependent variables, while partial mediation implies that the mediating variable explains some, but not all, of the link between the independent and dependent variables.

**Table 8:** Results of indirect effects.

**S/R**	**Hypothesis**	**Direct beta without mediation**	**Direct beta with mediation**	**Indirect beta/standardized estimates**	**Mediation type observed**
H^9^	Corporate cynical Impression → Economic & Legal CSR → Corporate Image	0.23^***^	0.19^***^	0.033	Partial Mediation
H^10^	Corporate cynical Impression → Philanthropic CSR → Corporate Image	0.23^***^	0.19^***^	0.278	Partial Mediation
H^11^	Corporate cynical Impression → Ethical CSR → Corporate Image	0.23^***^	0.19^***^	0.042	Partial Mediation
H^12^	Corporate cynical Impression → Economic & Legal CSR → Consumer Purchase Intention	0.17**	0.14^***^	0.016	Partial Mediation
H^13^	Corporate cynical Impression → Philanthropic CSR → Consumer Purchase Intention	0.17**	0.14^***^	0.158	Partial Mediation
H^14^	Corporate cynical Impression → Ethical CSR → Consumer Purchase Intention	0.17^**^	0.14^***^	0.030	Partial Mediation

*** *, Significance level at 0.001*.

** *, Significance level at 0.05*.

## Discussion

Company social responsibility has evolved into a critical component of corporate value generation and long-term operations and a globally recognized value. International organizations have produced applicable guidelines, such as the UN Global Compact and the OECD Guidelines for Multinational Enterprises, to assist firms in integrating CSR into their operational strategy and establishing organizational values for society. Numerous studies demonstrate that CSR has a beneficial influence on financial performance and may help organizations increase customer happiness and retention and improve the quality of products or services and enhance consumer purchase intention. As a result, this can help strengthen organizational cohesiveness and employee loyalty while also increasing work efficiency and reducing the corporate cynical impression. Thus, many experts argue that incorporating CSR into the fundamental beliefs of an organization may aid in achieving a competitive edge and sustainable operations. The firms can undoubtedly benefit their corporate image if they can fulfill their social duties. According to some marketing professionals, the function firms play in society affects perceptions of customers on corporate image and could also enhance consumer purchase intention by reducing the corporate cynical impression. Thus, how firms treat their employees, owners, and neighborhood citizens contributes to the image of the corporation toward their consumers. In addition, as the globe becomes more globalized, company success or failure is contingent on leveraging good CSR activities, and increasing customer buy intentions. CSR has a direct effect on consumer behavior according to the studies. As a result, “society first” has become the overarching principle for many organizations. Customer service and customer complaint centers are critical departments within businesses; any behaviors that annoy consumers are magnified and evaluated, even becoming significant performance indicators. It can be concluded that CSR is vital for companies.

Previous research on CSR has primarily focused on the influence of CSR on the financial success and employee behavior of a company, with little attention paid to the impact of CSR on corporate image and purchase intention of a consumer following the corporate cynical impression. Thus, the purpose of this study is to evaluate the links between corporate cynical impression, all three types of CSR, corporate image, and consumer purchase intentions.

## Conclusion and Recommendations

The corporate cynical impression is a serious issue for any kind of organization that is doing business in consumer markets. An organization could face a lot of issues which could include a bad image, propaganda, harsh customer feedback, and much more. It could negatively impact image of the company and consumer purchase intention. To counter the corporate cynical impression an organization could invest upon the factor of CSR. However, there are at least three kinds of CSR. If an organization has a low budget, then it could only invest in one type of CSR to control its operational cost. As the study results clearly showed that all hypotheses are statistically significant, and they also show the behavior of partial mediation, we need to recommend only that type of CSR that has the highest positive impact on corporate image and consumer purchase intention.

The results of the study that Philanthropic CSR could be considered as the most effective CSR type to reduce the cynical impression on corporate image and consumer purchase intention. Although the other two types are also statistically significant, they do not have that strong impact on both dependent variables as compared to the Philanthropic CSR. That is why it is recommended for organizations that if they want to fight with their cynical impression and also have a low budget and could invest only in one type of CSR, then they should invest in Philanthropic type of CSR for better corporate image and enhanced consumer purchase intention.

The need for time and resources could pose a significant obstacle to this research. When doing future studies, it must be kept in mind that these findings pertain directly to the individuals listed as respondents. To make it more accurate, they must be looked at in other demographic, psychographic, and geographies. It is therefore likely that when the same data is obtained from the same demographic that the findings will differ. To ensure more lasting and repeatable corporate outcomes, the studies would be required to conduct several times to obtain more accurate and reliable results influencing population and different factors.

## Data Availability Statement

The original contributions presented in the study are included in the article/Supplementary Material, further inquiries can be directed to the corresponding author.

## Ethics Statement

Ethical approval for this study and written informed consent from the participants of the study were not required in accordance with local legislation and national guidelines.

## Author Contributions

AARNA conceived and designed the concept literature review, data collection, and wrote the paper. YC and XW helped to provide technical support and contributed to analysis tools. JM and TRSM has reviewed the work to improve the outcomes. All authors have read and agreed to the published version of the manuscript.

## Conflict of Interest

The authors declare that the research was conducted in the absence of any commercial or financial relationships that could be construed as a potential conflict of interest.

## Publisher's Note

All claims expressed in this article are solely those of the authors and do not necessarily represent those of their affiliated organizations, or those of the publisher, the editors and the reviewers. Any product that may be evaluated in this article, or claim that may be made by its manufacturer, is not guaranteed or endorsed by the publisher.
